# Affordable miniaturized speckle contrast diffuse correlation tomography device for depth-sensitive mapping of cerebral blood flow in rodents

**DOI:** 10.1117/1.JBO.30.10.106007

**Published:** 2025-10-24

**Authors:** Fatemeh Hamedi, Faezeh Akbari, Mehrana Mohtasebi, Chong Huang, Li Chen, Lei Chen, Guoqiang Yu

**Affiliations:** a University of Kentucky, Department of Biomedical Engineering, Lexington, Kentucky, United States; b Bioptics Technology LLC, Advanced Science & Technology Commercialization Center (ASTeCC), Lexington, Kentucky, United States; c University of Kentucky, Department of Biostatistics, Lexington, Kentucky, United States; d University of Kentucky, Department of Neurosurgery, Lexington, Kentucky, United States

**Keywords:** depth sensitive, diffuse optical speckle imaging, cerebral blood flow, neuroscience research, rodents

## Abstract

**Significance:**

Continuous and longitudinal monitoring of cerebral blood flow (CBF) is critical for understanding brain pathophysiology and guiding interventions. Although rodents are the primary models in neuroscience, existing imaging modalities often fail to provide the optimal combination of low cost, high spatiotemporal resolution, wide head coverage, and sufficient penetration depth for small-animal brain imaging.

**Aim:**

Leveraging a clinical speckle contrast diffuse correlation tomography (scDCT) system, we aimed to develop an affordable, user-friendly, fast, and miniaturized scDCT (mini-scDCT) device tailored for depth-sensitive CBF imaging in small rodents.

**Approach:**

The mini-scDCT replaces bulky and costly optoelectronic components with compact, low-cost alternatives while preserving imaging performance. It is mounted on a standard stereotaxic apparatus for portability and ease of use. Temporal resolution was improved by hardware synchronization and software optimization. System validation was performed using head-simulating phantoms and rodent models under various pathophysiological conditions.

**Results:**

Compared with the original scDCT, the mini-scDCT achieved a fourfold cost reduction, a fivefold footprint reduction, and eightfold improvement in temporal resolution per source. Validation experiments confirmed the system’s depth sensitivity in head-simulating phantoms and its ability to detect both global and regional CBF changes in rodents, with results consistent with physiological expectations and prior studies.

**Conclusion:**

The mini-scDCT offers an affordable, user-friendly, depth-sensitive platform for functional brain imaging in rodent models. Its reduced cost and compact footprint enhance accessibility, whereas the improved spatiotemporal resolution enables diverse applications such as imaging brain functional connectivity in neuroscience research.

## 1 Introduction

Adequate cerebral blood flow (CBF) is essential for delivering oxygen and nutrients and removing metabolic waste to maintain normal brain function. Hypoperfusion reduces oxygen and nutrient delivery, leading to tissue ischemia and neuronal death, whereas hyperperfusion can cause excessive vascular pressure, increasing the risk of blood-brain barrier disruption and intracranial hemorrhage.^1^^,^^2^ Continuous and longitudinal monitoring of CBF is crucial for understanding cerebral pathological conditions and developing effective interventions.^3^^,^^4^

Rodents (mice and rats) have been used in neuroscience for more than a century and still make up 95% of animal models used in biomedical research today.^5^^–^^10^ However, a major limitation of neuroscience research in rodent models is the lack of affordable, portable, noninvasive imaging technologies that enable continuous, longitudinal monitoring of CBF variations across large brain regions. Large imaging modalities such as computed tomography, positron emission tomography, and magnetic resonance imaging (MRI) require expensive instrumentation and are difficult to use for continuous and longitudinal monitoring in standard preclinical or laboratory settings.^10^^–^^12^

Several minimally invasive imaging techniques have been explored for CBF monitoring in rodent models. These modalities generally involve trade-offs among spatial resolution, temporal resolution, penetration depth, and brain coverage.^13^ Contact-based functional ultrasound typically provides high spatiotemporal resolution for axial brain imaging but lacks whole-brain coverage.^14^^–^^16^ Similarly, contact wearable functional optical coherence tomography primarily maps cortical microvasculature with shallow penetration and limited field of view, limiting applications to superficial cortex or retinal imaging.^17^^,^^18^ Noncontact widefield optical methods, such as conventional or multiexposure laser speckle contrast imaging (LSCI) technologies, provide high spatiotemporal resolution with large brain coverage but are limited to superficial cortical layers.^19^^–^^23^ All these techniques require a craniotomy or skull thinning, which can alter the brain environment and hinder longitudinal CBF monitoring.^13^

To address these challenges, we have developed an innovative imaging technique known as speckle contrast diffuse correlation tomography (scDCT, US Patent #9861319). This approach enables noncontact, depth-sensitive, high-density 2D/3D imaging of CBF distributions at depths up to 10 mm and has been successfully applied to studies involving rodents,^24^^,^^25^ neonatal piglets,^26^^,^^27^ and human neonates.^28^ In the original scDCT system, a galvo mirror is used to rapidly (<1  ms switching time) and remotely direct focused, coherent, point, near-infrared (NIR) light to multiple source positions within a selected region-of-interest (ROI) on the head for deep brain penetration. A sCMOS camera is synchronized with the galvo mirror and captures sequential diffuse speckle contrast images at multiple source positions within the ROI. By adjusting the number of scanned sources, different ROI sizes can be covered. Small ROIs are suitable for rodents ($∼15\times 15\;{\mathrm{mm}}^{2}$ for mice and $∼25\times 25\;{\mathrm{mm}}^{2}$ for rats),^24^^,^^25^ whereas larger ROIs can be used for human neonates ($∼50\times 50\;{\mathrm{mm}}^{2}$).^28^ For example, ∼100 sources are typically sufficient for rodent imaging, whereas ∼500 sources may be needed to image larger ROIs in human neonates with adequate spatial resolution. Blood flow maps at different depths are reconstructed by incorporating boundary measurements of diffuse laser speckle contrasts on detection areas defined at specific distances from the scanning sources. The imaging depth is approximately one-third to one-half of the source detector (SD) separation.^29^ scDCT enables noninvasive, depth-sensitive CBF imaging, providing a powerful tool for investigating cerebral dynamics in both preclinical and clinical settings. Although effective, current scDCT systems are designed for broad use across both small and large subjects, including animals and humans. As a result, they are limited by bulky instrument dimensions, high cost, reduced maneuverability in specific applications (e.g., imaging small rodents), poor temporal resolution, and relatively complex operation.^24^^–^^28^

This paper presents our recent development of an affordable, user-friendly, fast, miniaturized speckle contrast diffuse correlation tomography (mini-scDCT) device, specifically designed for CBF imaging in small rodents. The mini-scDCT represents a significant advancement over the original large scDCT system, transitioning from an expensive, bulky, and cart-based setup to an inexpensive, compact, table-top implementation mounted on a standard small animal stereotaxic apparatus. Key advancements include the miniaturization of major components and the replacement of costly sCMOS cameras and galvo mirrors with more affordable alternatives, substantially reducing the device’s overall size and cost. In addition, the mini-scDCT was optimized to improve its temporal resolution while balancing its spatial resolution. The performance of the mini-scDCT was evaluated using 3D-printed head-simulating phantoms with known optical and geometric properties, as well as in rodents with pathological challenges to the brain. The results confirmed that the affordable mini-scDCT yields consistent findings with those obtained from other imaging modalities, including the larger and more expensive original scDCT system.

## 2 Methods and Materials

### 2.1 Development of an Affordable and Portable Mini-scDCT System for Rodents

#### 2.1.1 Original large and costly scDCT system

**Fig. 1:**
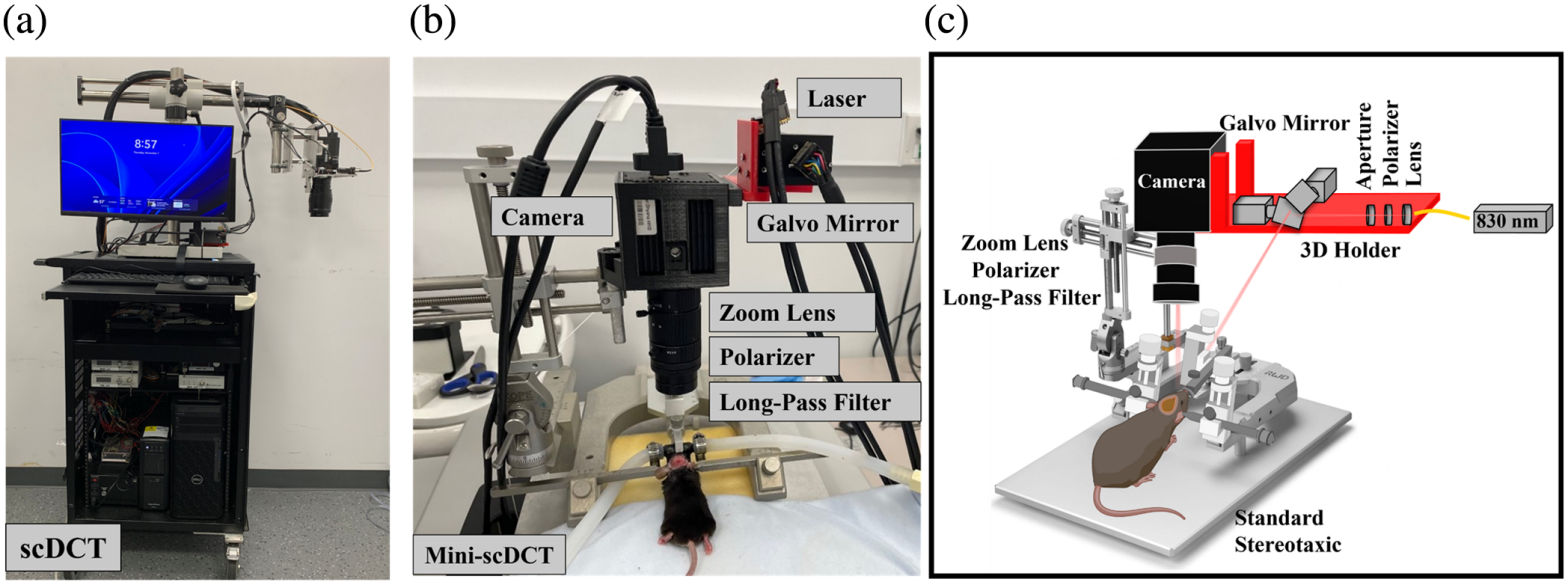
Two scDCT systems. (a) A large and expensive scDCT system sitting on a movable cart. (b) A compact and affordable mini-scDCT sitting on a table. (c) Schematic of the mini-scDCT with optoelectronic components.

In our original scDCT system [Fig. 1(a)], a galvo mirror (maximum scanning angle: ±12.5  degrees; angle step response: 300  μs; GVS002, Thorlabs, Newton, New Jersey, United States) delivers point NIR light, generated by a fiber-coupled long-coherence laser (wavelength: 785 nm; power: 150 mW, CrystaLaser, Reno, Nevada, United States), to multiple source positions.^25^ The point light was focused on the tissue surface via an achromatic lens (AC127-019-B, Thorlabs), and an aperture (SM05D5, Thorlabs) was used to control the spot size of the laser on the tissue. A large sCMOS camera (pixels: 2048×2048; bit depth: 16; quantum efficiency: 55% at 785 nm and 40% at 830 nm; frame rate: 30 fps, ORCA-Flash4.0, Hamamatsu Photonics, Hamamatsu, Japan) is used to sequentially capture diffuse spatial speckle contrast images at multiple source positions within a selected ROI.

A zoom lens (Zoom 7000, Navitar, Rochester, New York, United States) is connected to the camera for adjusting the camera’s focus on the ROI. A long-pass filter (cut-off wavelength: >750  nm; #84-761, EdmundOptics, Barrington, New Jersey, United States) is used to minimize the influence of ambient light during measurements. A pair of polarizers (LPNIRE050-B and LPNIRE200-B, Thorlabs) crossing the source and detection paths is added to reduce the specular reflection directly from the scanning light sources on the tissue surface. The fully noncontact scDCT probe is securely mounted on a rotating holder for easy alignment and adjustment. All optoelectrical components for the scDCT are assembled on a mobile cart.

The existing bulky scDCT system was designed as a multiscale device capable of imaging blood flow distributions across large tissue areas (up to $150\times 150\;{\mathrm{mm}}^{2}$) and great tissue depths (up to centimeters).^27^ Consequently, it incorporates costly, large, and high-quality optoelectronic components. However, such specifications exceed the requirements for imaging small rodent heads, which require relatively smaller ROIs (e.g., $15\times 15\;{\mathrm{mm}}^{2}$ for mice and $25\times 25\;{\mathrm{mm}}^{2}$ for rats) and shallower penetration depths (e.g., 1 to 3 mm for rodents). In addition, the system lacks user-friendliness, demands specialized instruction for operation. Furthermore, its low temporal resolution (e.g., 0.2 s per source^26^) limits applicability in scenarios requiring high-speed imaging.

#### 2.1.2 Transition to an affordable and portable mini-scDCT system

To miniaturize the system while maintaining comparable performance, optoelectronic components in both the source and detection paths were replaced with smaller, cost-effective alternatives [Figs. 1(b) and 1(c)]. In the source path, the galvo mirror was replaced with a compact model (Compact-506, maximum scanning angle: ±25  deg, angle step response: 150  μs, ScannerMax, Sanford, Florida, United States). The new galvo mirror doubled the scanning speed, improving temporal resolution while reducing costs by ∼50%.

In the detection path, the ORCA-Flash 4.0 camera (dimensions: $85.5\times 85.5\times 95.3\;{\mathrm{mm}}^{3}$; weight: 1100 grams, Hamamatsu) was replaced with a smaller, faster, and more affordable Dhyana 401D camera (dimensions: $56.0\times 56.0\times 61.7\;{\mathrm{mm}}^{3}$; weight: 305 grams, Tucsen, Fuzhou, Fujian, China). Despite significant reductions in size, weight, and cost (at approximately one-third the price), the Dhyana 401D camera delivered comparable or superior performance (pixels: 2048×2048; bit depth: 16; quantum efficiency: 50% at 785 nm and 45% at 830 nm; frame rate: 40 fps) to the ORCA-Flash 4.0 camera.

In addition, other optical components with two-inch diameters, including a polarizer, a zoom lens, and an optical filter, were replaced with equivalent 1-in diameter components. This modification further reduced the device’s size and cost without compromising performance. Table 1 lists key components used to assemble the original scDCT and mini-scDCT devices, along with their overall cost and dimensions. These optimizations yielded a fourfold cost reduction and a fivefold smaller footprint for the mini-scDCT.

**Table 1:** Comparison between key parts/components for assembling the original scDCT and mini-scDCT devices.

System	Illumination		Detection				Instrumentation	
	Source	Galvo mirror	Camera	Zoom lens	Polarizer	Long-pass filter	Cost ($)	Dimension (inches)
scDCT	785 nm	GVS002	Orca-Flash 4.0	7000	LPNIRE200-B	>750 nm	∼35 k	20 × 20 × 24
						#84 to 761		
	CrystaLaser	Thorlabs	Hamamatsu	Navitar	Thorlabs	Edmund Optics		
Mini-scDCT	785 or 830 nm	Compact-506	Dhyana 401D	3.3× Macro	LPNIRE100-B	>600 nm	∼10 k	12 × 10 × 14
						FELH0600		
	CrystaLaser	Scanner Max	Tucsen	Computar	Thorlabs	Thorlabs		

#### 2.1.3 Temporal resolution improvement

The original scDCT system had a low temporal resolution (e.g., 0.2 s per source^26^). Attempts to increase temporal resolution led to frame loss due to limited camera acquisition speed and storage. Data could be saved either to RAM or directly to the hard drive: RAM allowed high temporal resolution for short recordings but quickly exceeded capacity during long acquisitions, causing instability, whereas hard-drive storage avoided this risk but reduced temporal resolution. To prevent frame loss, a trigger signal initiated image capture at each galvo position, though saving delays limited the overall sampling rate.

To improve the temporal resolution, several key modifications were implemented in the mini-scDCT system. First, a new Compact-506 galvo mirror with a response time twice as fast as the original one was installed, reducing mechanical delays and enabling quicker scanning of source positions. Second, the Dhyana 401D camera, offering a 1.3× frame rate and onboard buffer memory, was implemented to temporarily store multiple frames before transferring them to the computer, preventing frame loss. Third, the LabVIEW code was redesigned. Originally, it generated a trigger signal at each scanning position via a DAQ module (Measurement Computing USB-3101) to reposition the galvo mirror. In the new design, an additional loop was added to read the camera’s trigger signal and use it to control the galvo mirror. This approach enabled precise synchronization without requiring predefined inter-frame delays. These changes reduced latency and allowed reliable high-speed synchronization. As a result, the mini-scDCT achieved an eightfold improvement in temporal resolution. Acquisition time per source decreased from 0.2 to 0.025 s. For example, 100 sources could now be scanned in 2.5 s instead of 20 s. This improvement supports high-speed applications such as detecting low-frequency oscillations at 0.01 Hz for studying brain functional connectivity, which requires a sampling time of <5  s.^26^^,^^30^

#### 2.1.4 Operational efficiency enhancement

The existing scDCT system requires specialized instruction for operation and involves configuring multiple parameters tailored to specific experimental setups. Moreover, the large rotating probe holder, mounted on a heavy arm, complicates adjustment of the working distance and ROI, limiting setup flexibility. This is particularly problematic when imaging small animals, such as rodents, where precise alignment to a small ROI of 15 to 30 mm is essential. These limitations pose significant challenges for longitudinal studies that demand accurate repositioning across multiple time points.

To overcome these challenges, the new mini-scDCT features a customized holder designed in SOLIDWORKS and fabricated using a 3D printer (X-MAX, QIDI 3D). All optical components in the source path are integrated into the compact holder and optimally aligned with the detection path, eliminating the need for manual illumination alignment [Fig. 1(b)]. The resulting probe is mounted onto a commercial stereotaxic platform (900LS, KOPF) for stable fixation of the rodent’s head, enhancing portability and ease of use. This unique design ensures consistent animal positioning across repeated measurements by maintaining the same working distance, magnification, and galvo mirror alignment. Once configured for a subject, typically no readjustment is required for subsequent sessions, allowing repeated imaging with the same setup for a given ROI size. If repositioning causes significant shifts, the acquired images are aligned using the bregma as the central reference point.

The customized LabVIEW code and updated camera software were optimized for user-friendliness, featuring a redesigned graphic user interface that allows device control with just a few control buttons. Source scanning and essential functions are now accessible directly from the main interface, minimizing setup time, reducing errors, and enhancing usability. As a result, researchers with no prior experience can operate mini-scDCT by simply following the step-by-step instructions in the operation manual.

### 2.2 Head-Simulating Phantom Experiments

**Fig. 2:**
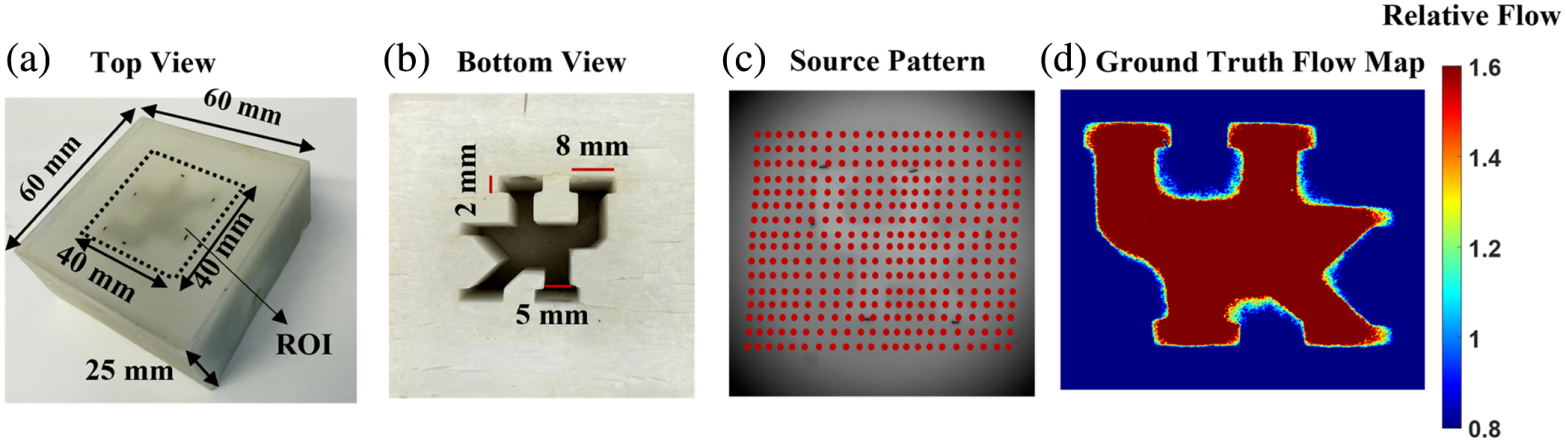
Head-simulating phantoms with known optical and geometric properties. (a) Top view of the head-simulating phantoms with a top layer thickness of 1 mm. The dashed square indicates the scanning ROI of $40\times 40\;{\mathrm{mm}}^{2}$. (b) Bottom view of the solid phantom with the empty UK logo channels. (c) The distribution of 400 sources within the selected ROI. (d) The ground truth flow map obtained by imaging the UK logo phantom flipped upside down.

Tissue-simulating phantoms with known optical and geometric properties are widely used to validate diffuse optical imaging technologies. Following our established methods,^25^^,^^31^ 3D-printed layered phantoms were fabricated to mimic the skull and brain tissues for evaluating the depth sensitivity of mini-scDCT in flow contrast mapping (Fig. 2). The design included a solid surface skull layer (no flow) and a liquid Intralipid solution (with particle flow), with the solid layer featuring University of Kentucky (UK) logo-shaped channels and fabricated using a Prusa SL1 printer.

Three head-simulating phantoms were fabricated with the top layer thicknesses of 1, 2, and 3 mm, respectively [Figs. 2(a) and 2(b)].^25^ The solid phantom was prepared using the following recipe to achieve the expected tissue optical properties. The formulation consisted of 0.4 g titanium dioxide (${\mathrm{TiO}}_{2}$), 0.27 mL black India ink (Massachusetts), 7 mL dimethyl sulfoxide (DMSO), and 180 mL clear resin (eSUN, Hard-Tough). These components were thoroughly mixed and used to fill the 3D printer container for phantom fabrication. For the liquid phantom, a mixture of 2.1 mL Intralipid solution (Fresenius Kabi, Sweden), 0.01 mL India ink, and 47 mL distilled water was mixed to fill the channels of the printed structures. After filling, the UK-shaped channels were sealed with plastic film and hot-melt adhesive to prevent leakage.

The solid and liquid phantoms were matched in optical properties, with an absorption coefficient $\mu_{a}$ of $0.03\;{\mathrm{cm}}^{-1}$ and a reduced scattering coefficient $\mu_{s}^{\prime }$ of $9\;{\mathrm{cm}}^{-1}$. The optical properties of both phantoms were experimentally verified using a commercial frequency-domain near-infrared spectroscopic system (Imagent 049-SP, ISS Medical).^25^^,^^32^ Intralipid particle Brownian motion simulated red blood cell dynamics (i.e., blood flow). Because this motion is temperature-dependent, the liquid phantom temperature was continuously monitored and controlled with a thermosensor (TR75A2, TandD) to ensure experimental consistency.

The mini-scDCT scanned 400 source positions (20×20) over a ROI of $40\times 40\;{\mathrm{mm}}^{2}$ on the phantom surface [Fig. 2(c)], balancing spatial and temporal resolutions as well as computation efficiency. The camera operated at a frame rate of 35 fps with an exposure time of 5 ms. Scanning of the ROI with 400 sources resulted in a system temporal resolution of ∼10  s (i.e., 400 sources × 0.025 s per source). The optical setting (0.3× magnification, f/8) produced a 19.9  μm speckle size,^33^ which is well above the 6.5  μm camera pixel size, satisfying the Nyquist criterion for alias-free imaging.^34^ The ground truth flow map [Fig. 2(d)] was obtained by imaging the UK logo phantom flipped upside down [Fig. 2(b)] using the mini-scDCT device. All measurements in phantoms and animals were performed in a dimly lit environment.

### 2.3 Animal Experiments

All experimental protocols on animals were approved by the UK institutional animal care and use committee (IACUC) under protocol No. 2016-2508. *In vivo* experiments were conducted in nine mice with ischemic stroke or sham surgeries to evaluate the mini-scDCT’s capability for continuous monitoring of CBF variations over 24 h and in one rat subjected to ${\mathrm{CO}}_{2}$ inhalation and transient common carotid artery (CCA) ligation to assess the system’s ability to image through a thicker skull to the deeper brain.

#### 2.3.1 *In vivo* experiments in mice with ischemic stroke

A middle cerebral artery occlusion (MCAO) stroke model of mice was utilized to evaluate the mini-scDCT’s ability for continuous monitor of CBF variations over 24 h after brain infarct.^35^ Nine mice (C57/Bl6, male, 4 to 6 months old, 23-30 grams) were used: six underwent unilateral MCAO surgery, and three received sham surgeries without occlusion as controls (Table 2). Low concentration of isoflurane anesthesia (1% to 2%) was used in this study because it provided precise control, rapid onset and recovery, minimal stress, and fewer complications during prolonged imaging (e.g., 1 h) than injectable ketamine-Xylazine. After anesthesia, a midline neck incision was made, and the left CCA was isolated. A silicone-coated 5-0 nylon filament (Doccol, Sharon, Massachusetts, United States) was inserted into the internal carotid artery (ICA) through a small incision and advanced 12 mm to occlude the MCA at the Circle of Willis. The filament remained in place for 24 h to induce permanent ischemic stroke. Sham mice underwent the same procedure without ICA incision or MCAO.

**Table 2:** Subject information and experimental protocols.

Subjects	Age (months)	Experimental protocols	Source numbers
Mouse #1	5	Sham	20 × 20
Mouse #2	6	Sham	20 × 20
Mouse #3	4	Sham	20 × 20
Mouse #4	5	MCAO	20 × 20
Mouse #5	6	MCAO	20 × 20
Mouse #6	4	MCAO	20 × 20
Mouse #7	6	MCAO	20 × 20
Mouse #8	4	MCAO	20 × 20
Mouse #9	4	MCAO	20 × 20
Rat #1	8	8% ${\mathrm{CO}}_{2}$ inhalation, transient CCA ligation	10 × 10

On the surgery day (day 1), the anesthetized mouse was laid prone on a heating blanket, its head was secured on the stereotaxic frame, and the scalp was cut open to expose the skull for mini-scDCT imaging. Mini-scDCT imaging was performed intermittently at baseline (pre-surgery) and at 10-, 20-, 40-, and 60-min post-surgery. At each time point, two CBF images were acquired over a 20-s period and averaged to enhance signal quality. A total of 400 sources (20×20) were used to scan the head with an ROI of $15\times 15\;{\mathrm{mm}}^{2}$ at each time point with a system magnification of 0.5×. The camera was set to a frame rate of 35 Hz with an exposure time of 5 ms. Again, scanning of the ROI with 400 sources yielded a system temporal resolution of ∼10  s. After imaging, the scalp was sutured back, and the animal was allowed to return to its home cage to recover. At 24 h post-stroke (day 2) or sham surgery, neurological deficits were assessed via behavioral tests. Each mouse was gently lifted by the tail, and spontaneous motor responses were observed. Following re-anesthetization, the mouse’s scalp was re-opened, and mini-scDCT imaging was repeated.

#### 2.3.2 *In vivo* experiments in one rat during CO_2_ inhalation and transient CCA ligations

A case study on an adult rat (Wistar, male, 8-month-old, ∼650  g) was conducted to evaluate mini-scDCT’s ability to image through a thicker skull and reach deeper brain regions, compared with mice. ${\mathrm{CO}}_{2}$ inhalation and transient CCA ligation protocols were used for direct comparison with our previous scDCT studies in rats,^25^ with a wavelength of 785 nm applied to maintain consistency.

The rat was anesthetized with 2% to 3% isoflurane. Following a midline incision, both the left and right CCAs were isolated. A loose surgical suture loop was placed around each CCA without restricting blood flow. Mini-scDCT was performed continuously during a ${\mathrm{CO}}_{2}$ inhalation protocol consisting of a 5-min baseline, 5-min 8% ${\mathrm{CO}}_{2}$ inhalation via nose cone, and a 15-min recovery. After 30 min, the second imaging phase began and was performed continuously. Following a 3-min baseline recording, the left CCA was ligated by tightening the surgical suture on it for 5 min, followed by ligation of the right CCA to induce a transient global ischemia for 2 min. Then, the right ligation was released to restore CBF in the right hemisphere for 5 min, followed by releasing the left CCA ligation to restore CBF globally.

For continuous mini-scDCT imaging, the rat was placed in a prone position on a heating blanket, and its head was stabilized in a stereotaxic frame with the scalp retracted. A total of 100 source numbers (10×10) were used to enhance temporal resolution. The imaging system was configured with a magnification of 0.4× to cover an ROI of $20\times 20\;{\mathrm{mm}}^{2}$. The camera settings included a frame rate of 35 Hz and an exposure time of 5 ms. Scanning of the ROI with 100 sources resulted in a system temporal resolution of ∼2.5  s.

### 2.4 Mini-scDCT Data Analysis

#### 2.4.1 Depth-sensitive flow image reconstruction

The fast and depth-sensitive diffuse speckle contrast topography algorithm was used to reconstruct flow images, as described in our previous publication.^25^ Briefly, raw intensity images captured at each source position ($S_{i}$) were converted into spatial speckle contrast images using the following equation:

$$K_{s}(i)=\frac{\sigma }{\left\langle I\right\rangle }=\frac{\sqrt{\left(\langle I^{2}\rangle -\langle I\rangle^{2}\right)}}{\left\langle I\right\rangle },$$

where $K_{s}(i)$ represents the speckle contrast at source location $S_{i}$, calculated as the ratio of the standard deviation (σ) to the mean intensity (⟨I⟩) within a 7×7  pixel window. A sliding window was applied across all pixels within the ROI to generate a 2D map of $K_{s}(i)$.

To enhance computational efficiency, MATLAB’s built-in convolution functions and kernel matrices were utilized in this step. The MATLAB functions were described in our previous publications,^25^^,^^26^^,^^33^ and all data analysis was performed in MATLAB R2022a. Speckle contrast was calculated with a custom MATLAB script using MATLAB’s built-in 2D convolution function (conv2, Image Processing Toolbox™) with a 7×7 uniform kernel.

**Fig. 3:**
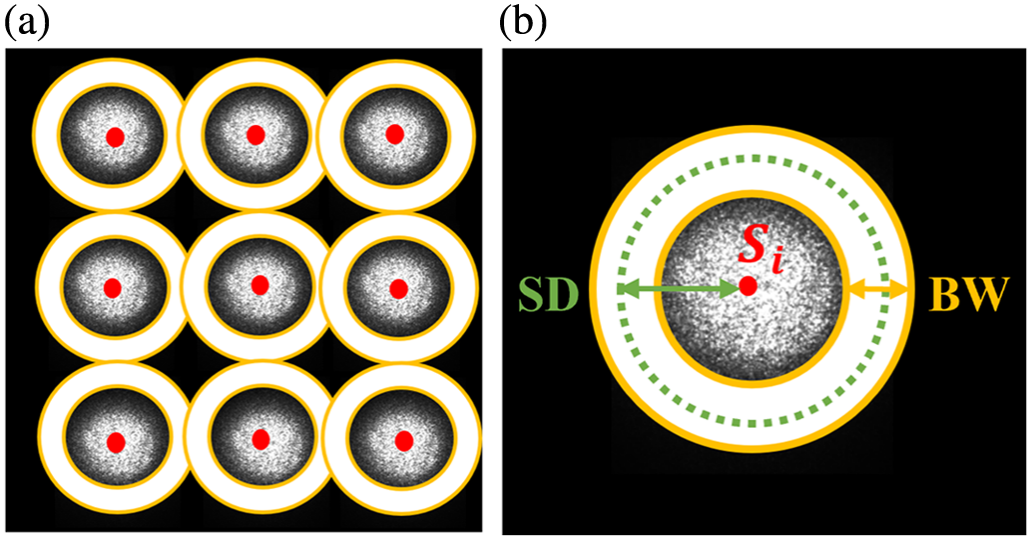
Illustrative source-detector pattern with definitions of $S_{i}$, SD, and BW. (a) A 3×3 source pattern with detectors defined around each point source. Solid red points mark the source centers identified using Otsu’s method. (b) Example of an SD mask for a representative source $S_{i}$, showing the SD separation (green arrow) and BW boundaries (yellow circles).

According to photon diffusion theory, the maximum penetration depth (PD) of diffusive light in tissue is approximately one-third to one-half of the SD separation.^29^ To extract a spatial speckle contrast map at a specific PD (i.e., $K_{\mathrm{PD}}$), the process began with identifying each source location $S_{i}$. As shown in Fig. 3(a), the intensity image was first converted to a binary image using Otsu’s method,^36^ which automatically determined the optimal threshold to separate bright and dark regions. Connected groups of white pixels were then identified, and the largest group was retained as the source position. The center and radius of each source were subsequently calculated, and this procedure was repeated for all source locations.

A binary belt-shaped mask was then defined around each source center, assigning a value of “1” to pixels within the belt width (BW) at a specified SD separation and “0” to all other pixels outside the detector belt [Fig. 3(b)]. This mask was multiplied by $K_{s}(i)$ and summed across all source locations to generate the corresponding $K_{\mathrm{PD}}$ map. To reduce artifacts caused by the summation of $K_{s}(i)$ in the overlap regions of masks [Fig. 3(a)], the inverse of mask summation was multiplied by $K_{s}(i)$ summation.

The blood flow index (BFI) map at a given depth (PD) was then calculated as $\mathrm{BFI}=1/K_{\mathrm{PD}}^{2}$. Relative time-course changes in CBF (rCBF) were calculated by normalizing BFI values at each time point to their respective baseline values obtained before pathophysiological interventions. This 2D mapping approach requires no specialized hardware, runs on a standard workstation for near-real-time data processing.

#### 2.4.2 Evaluation of peak signal-to-noise ratio in head-simulating phantom images

To evaluate the depth sensitivity of mini-scDCT, 2D flow maps reconstructed at different SD separations and with different BWs were evaluated using peak signal-to-noise ratio (PSNR), a standard metric for quantifying image reconstruction quality.^37^ PSNR is defined as $\mathrm{PSNR}=10\;\log_{10}(\frac{{\mathrm{MAX}}^{2}}{\mathrm{MSE}})$, where MAX is the maximum BFI value in the ground truth image [Fig. 2(d)], and MSE is the mean squared error between the ground truth and reconstructed BFI values.

#### 2.4.3 Quantification of mini-scDCT spatial resolution in rodents

Following our established method,^31^ spatial resolution was quantified by selecting ROIs of varying sizes over different cerebral vessels in BFI maps of mice and rats. Vessel diameters were then estimated from the pixel counts within each ROI, scaled to the corresponding head dimensions, thereby representing the spatial resolution of mini-scDCT in rodent imaging.

#### 2.4.4 Extraction of head geometry in rodents

As detailed in our previous study,^38^ head surface geometry was extracted by summing all raw intensity images taken at all source positions to create a composite intensity map. A Gaussian filter (kernel size: 20) was applied to suppress high-frequency speckle noise, with smoothing levels adjustable to balance noise reduction and spatial detail. Next, a height map was generated based on the normalized intensity values at each pixel. The intensity values were divided into 256 bins, each corresponding to a specific height level, with higher intensities indicating greater surface elevations. Finally, the 2D flow map was overlaid onto the 3D height map to visualize both head geometry and blood flow, enabling more intuitive interpretation of anatomical cerebral hemodynamics. This visualization also facilitated the alignment of multiple images acquired repeatedly over the 24-h period.

#### 2.4.5 Statistical data analysis

The repeated measures ANOVA was used to compare rCBF across different time points within the stroke and sham groups separately. Mauchly’s test of sphericity was first conducted to assess the assumption of sphericity. If Mauchly’s test was not significant, the sphericity-assumed test was used to calculate the overall p-value; otherwise, the Greenhouse-Geisser test was applied. Furthermore, if the overall p-value was significant, post hoc pairwise comparisons were performed to identify the pair of time points with significant differences. In addition, the two-sided two-sample t-test was used to compare rCBF between the stroke and sham groups at each time point. Levene’s test was conducted to assess the assumption of equal variance. If Levene’s test was not significant, the t-test assuming equal variance was used; otherwise, the t-test without assuming equal variance was used. Data analyses were performed using SPSS software, and results with p<0.05 were considered significant.

## 3 Results

### 3.1 Mini-scDCT Enabled Depth-Sensitive Mapping of Head Simulating Phantoms

**Fig. 4:**
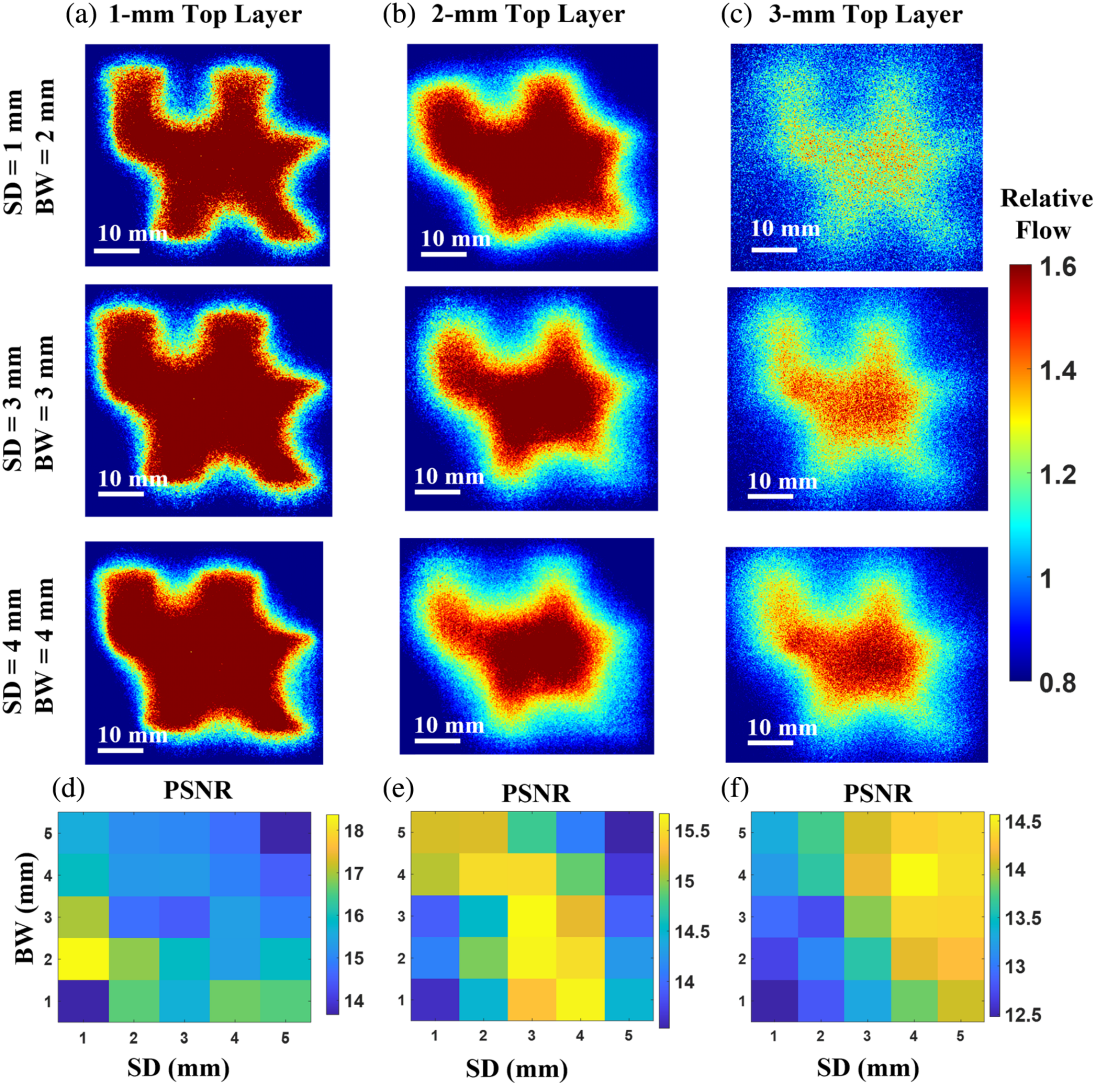
Depth-sensitive mapping of Intralipid particle flow in head-simulating phantoms. (a)–(c) Resulting 2D maps of Intralipid particle flow contrasts on three head-simulating phantoms with the top layer thicknesses of 1, 2, and 3 mm, respectively. Flow indices were normalized to their mean value to generate relative flow maps for comparisons. (d)–(f) The PSNR distributions with varied SDs (1 to 5 mm) and varied BWs (1 to 5 mm) for the head-simulating phantoms with top layer thicknesses of 1, 2, and 3 mm, respectively.

Figures 4(a)–4(c) show 2D flow maps reconstructed with varying SDs and BWs from the UK logo phantoms with the top layer thicknesses of 1, 2, and 3 mm, respectively. These phantoms contain UK flow channels with a dimension ranging from 2 to 8 mm [Fig. 2(b)]. Figures 4(d)–4(f) present the PSNR results under varying SDs and BWs for all three phantoms. The ground truth flow map was obtained by imaging the UK logo phantom flipped upside down [Fig. 2(d)]. The higher the PSNR, the better the quality of the reconstructed images.

The highest PSNR values were observed differently for different phantoms using different configurations of SDs and BWs. For the phantom with a top layer thickness of 1 mm, the maximum PSNR value of 18 appeared with the SD of 1 mm and BW of 2 mm. For the phantom with a top layer thickness of 2 mm, the maximum PSNR value of 15.5 shifts to a larger SD of 3 mm and BW of 3 mm. For the phantom with a top layer thickness of 3 mm, the maximum PSNR value of 14.5 was obtained with the SD of 4 mm and BW of 4 mm. These findings are expected as deeper penetration with large SDs and thicker top layer resulting in fewer diffused photons being detected, thus leading to lower PSNRs. These results provide guidance for optimizing SDs and BWs to achieve the best image quality based on specific rodent head geometries.

### 3.2 Mini-scDCT Enabled Mapping BFI and Head Geometry in Mice

**Fig. 5:**
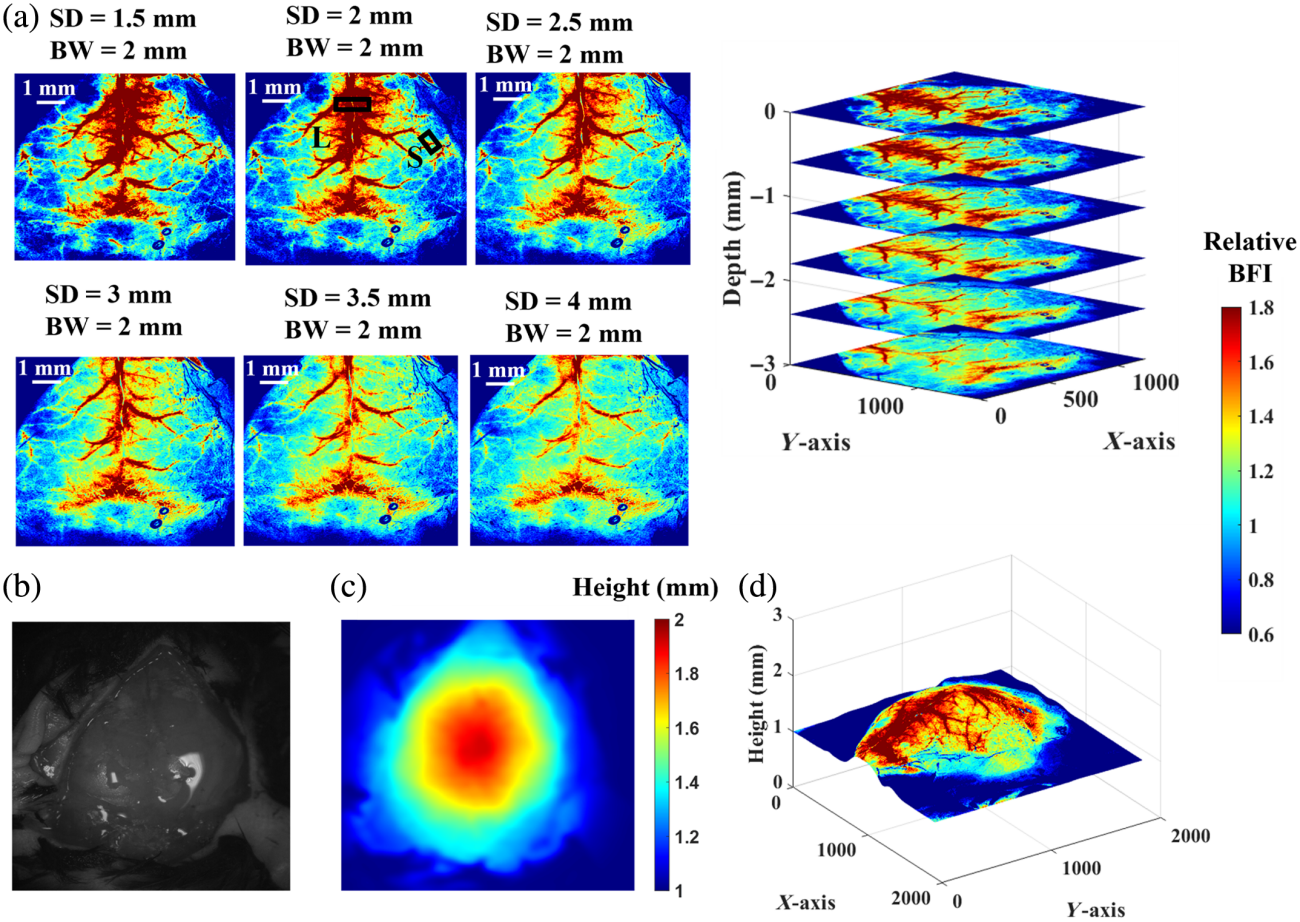
Depth-sensitive imaging of BFI maps from the head of a control mouse (Mouse #2, sham) at baseline. (a) Series of 2D BFI maps reconstructed using SDs ranging from 1.5 to 4 mm with BW = 2 mm. Spatial resolution was quantified by selecting two ROIs on the 2D flow map (SD = 2 mm and BW = 2 mm): one over a large bundle of superior cerebral veins (L) and another over a small vessel (S). (b) Photograph of the mouse skull. (c) Corresponding height map of the skull. (d) Integrated 2D BFI map overlaid on the 3D head surface geometry.

Figure 5(a) shows depth-sensitive BFI maps of a sham mouse (Mouse #2, Table 2) during the baseline measurement, illustrating the brain’s vascular structure at different depths. BFI values were normalized to their mean value, yielding relative BFI maps for comparisons. Given that the average adult mouse skull thickness is ∼0.3  mm,^39^ a series of 2D BFI maps were reconstructed using SDs ranging from 1.5 to 4 mm and a detection BW of 2 mm. These configurations corresponded to PDs of 0.5 to 2 mm. Figures 5(b) and 5(c) show a photograph and the corresponding height map of the mouse head (Mouse #2), respectively. Figure 5(d) presents the integrated BFI map overlaid on the 3D head surface geometry (reconstructed from the height map), aiding in the analysis of localized BFI variations.

Based on the results in Figs. 4 and 5, an SD of 2 mm and a BW of 2 mm were selected to capture cortical BFI at depths of 0.5 to 1 mm in mice. Spatial resolution was then quantified by selecting ROIs over a small vein (S) and a large vein (L) in Fig. 5(a), with estimated values of ∼40 and ∼360  μm, respectively, calculated from pixel counts within the ROIs and scaled to the corresponding head dimensions.

### 3.3 Mini-scDCT Enabled Longitudinal Monitoring of CBF Variations in Mice

**Fig. 6:**
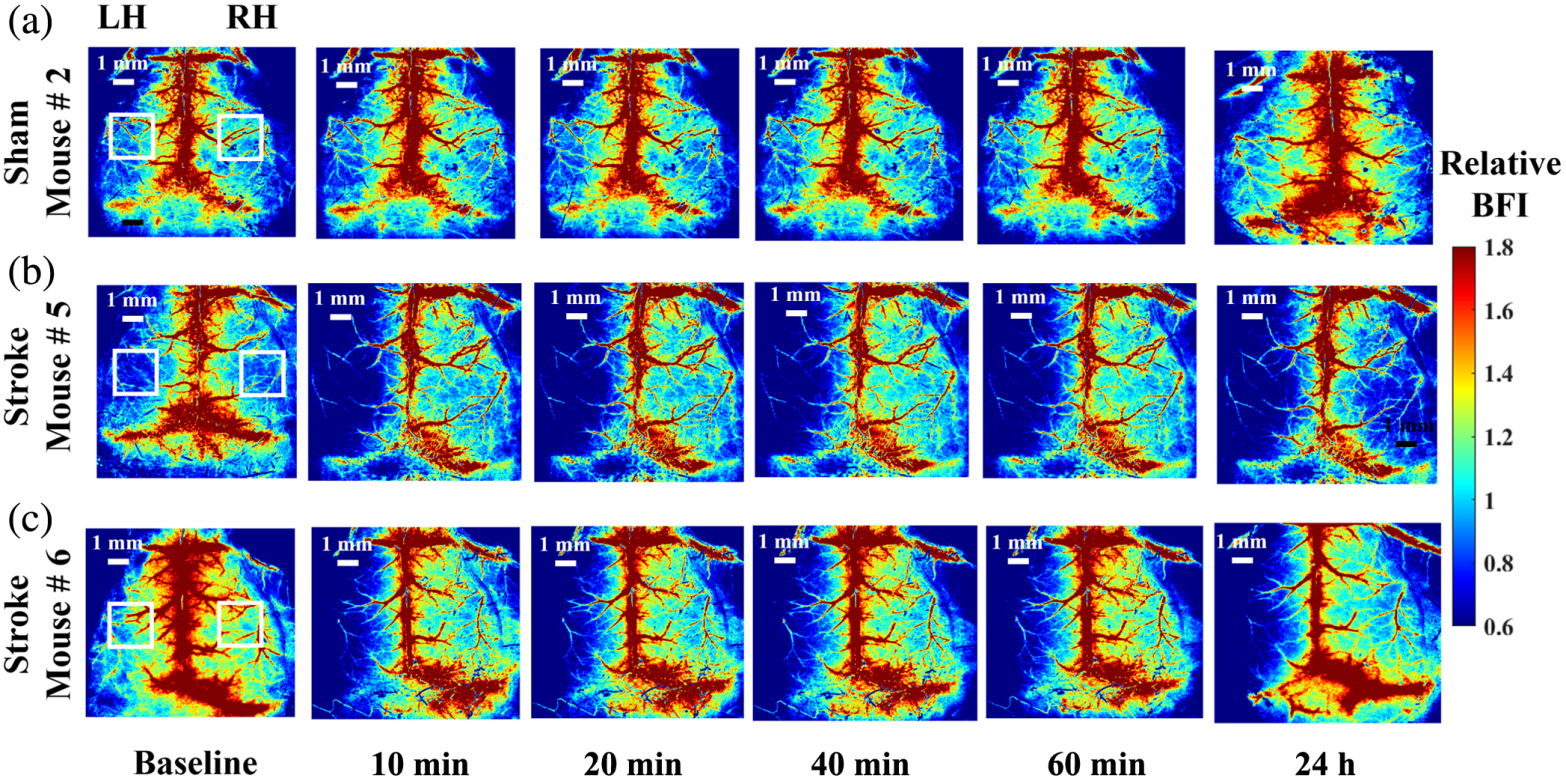
2D reconstructions of relative BFI maps at the baseline, 10, 20, 40, and 60 min and 24 h after surgery. (a)–(c) Relative BFI maps over time in Mouse #2, Mouse #5, and Mouse #6, respectively. The rectangular boxes on the LH and RH were selected to assess hemispheric differences in BFI changes over time.

Figures 6(a)–6(c) show BFI maps analyzed with an SD of 2 mm and a BW of 2 mm in representative mice: Mouse #2 (sham group) and Mouse #5 and Mouse #6 (stroke group, see Table 2). Two ROIs were placed on the left hemisphere (LH) and right hemisphere (RH) to assess hemispheric differences in BFI changes over time. rCBF values were calculated by normalizing the mean BFI of each hemisphere at each time point to its respective baseline value on day 1.

**Fig. 7:**
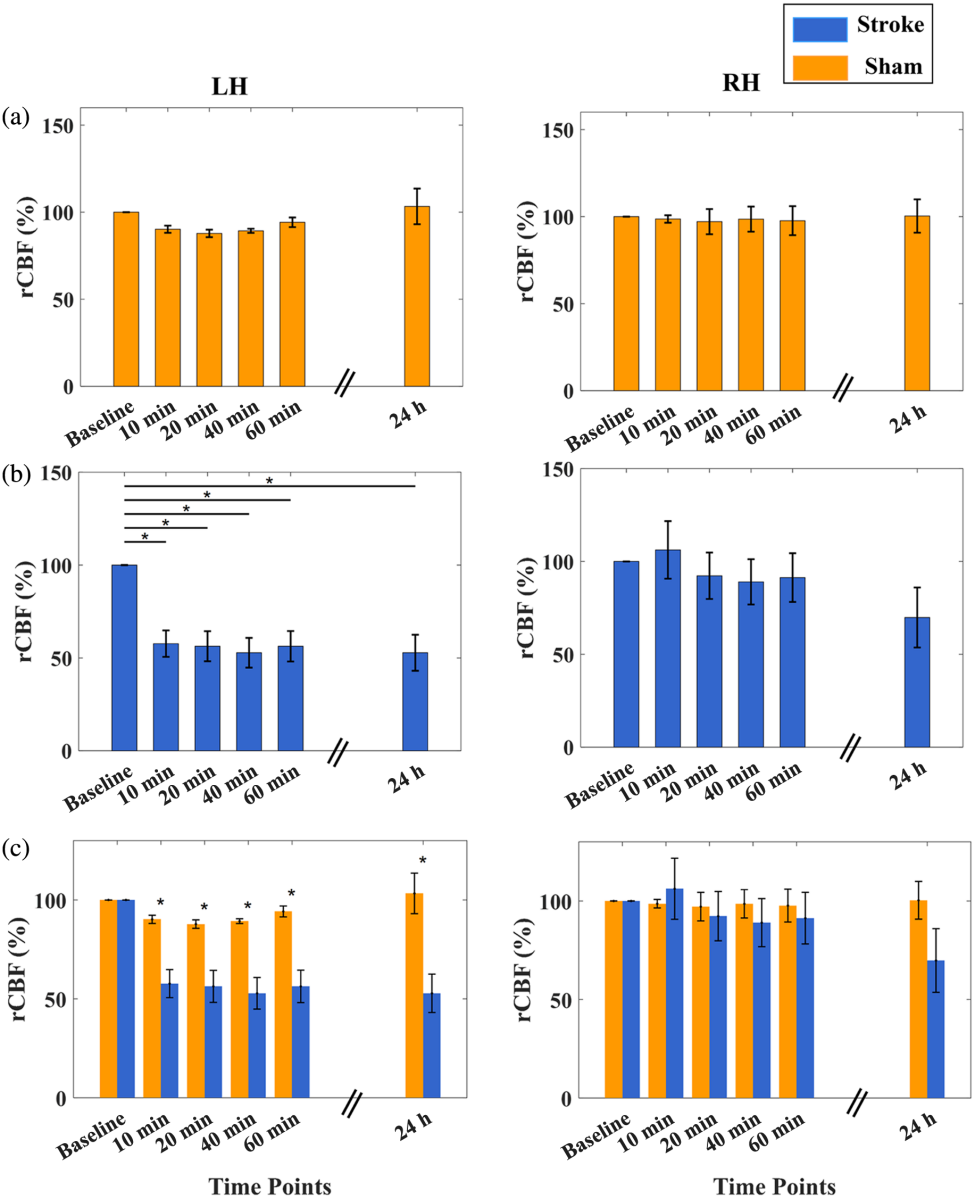
Longitudinal rCBF variations in both sham and stroke groups. (a) Sham Group: no significant changes over time were observed. (b) Stroke group: significant rCBF reductions over time in the left hemisphere with stroke were observed. (c) The between-group differences over time: significant rCBF differences between groups were observed in the left hemisphere with stroke. Star (*) denotes p<0.05.

Figure 7(a) shows the time-course rCBF variations in each hemisphere of the sham group (n=3). As expected, no significant changes in rCBF were observed in either hemisphere during the 60-min monitoring period on days 1 and 2, as evaluated by repeated measures ANOVA.

Figure 7(b) depicts the time-course changes in rCBF in each hemisphere of the stroke group (n=6). On day 1, rCBF in the LH showed an immediate decline following the induction of left-side MCAO and remained persistently low throughout the 60-min monitoring period. By contrast, rCBF in the RH exhibited a transient, slight increase within the first 10 min following stroke induction, followed by a gradual decline over the subsequent monitoring period. This early contralateral hyperemia likely reflects an autoregulatory or compensatory vascular response, wherein the intact hemisphere attempts to redistribute cerebral perfusion to mitigate the impact of unilateral ischemia. The subsequent decrease in rCBF suggests that the compensatory mechanism was insufficient to maintain elevated perfusion potentially due to global hemodynamic disturbances or impaired cross-hemispheric vascular communication in the setting of a permanent stroke.

On day 2, rCBF in the LH remained at the same reduced level observed on day 1, indicating sustained hypoperfusion and confirming the presence of a permanent stroke. Meanwhile, rCBF in the RH also declined to a level comparable to that of the LH, suggesting that the compensatory mechanism to maintain bilateral cerebral perfusion was lost 24 h post-stroke. Table S1 in the Supplementary Material quantifies time-course rCBF variations in two hemispheres for both groups.

Figure 7(c) illustrates the between-group differences over time, assessed using a two-sided two-sample t-test. The results shown in Fig. 7 are consistent with expected outcomes in a permanent MCAO model and further validate the observed hemodynamic changes.

Moreover, all stroke mice subjected to left-side MCAO exhibited characteristic post-stroke behaviors, including unilateral spinning or circling toward the affected side, well-established indicators of motor asymmetry, and qualitative confirmation of successful stroke induction. By contrast, all sham mice displayed normal behavior with no signs of motor asymmetry, reinforcing the specificity of the observed deficits in the stroke group.

### 3.4 Mini-scDCT Enabled Mapping BFI Variations in a Rat

**Fig. 8:**
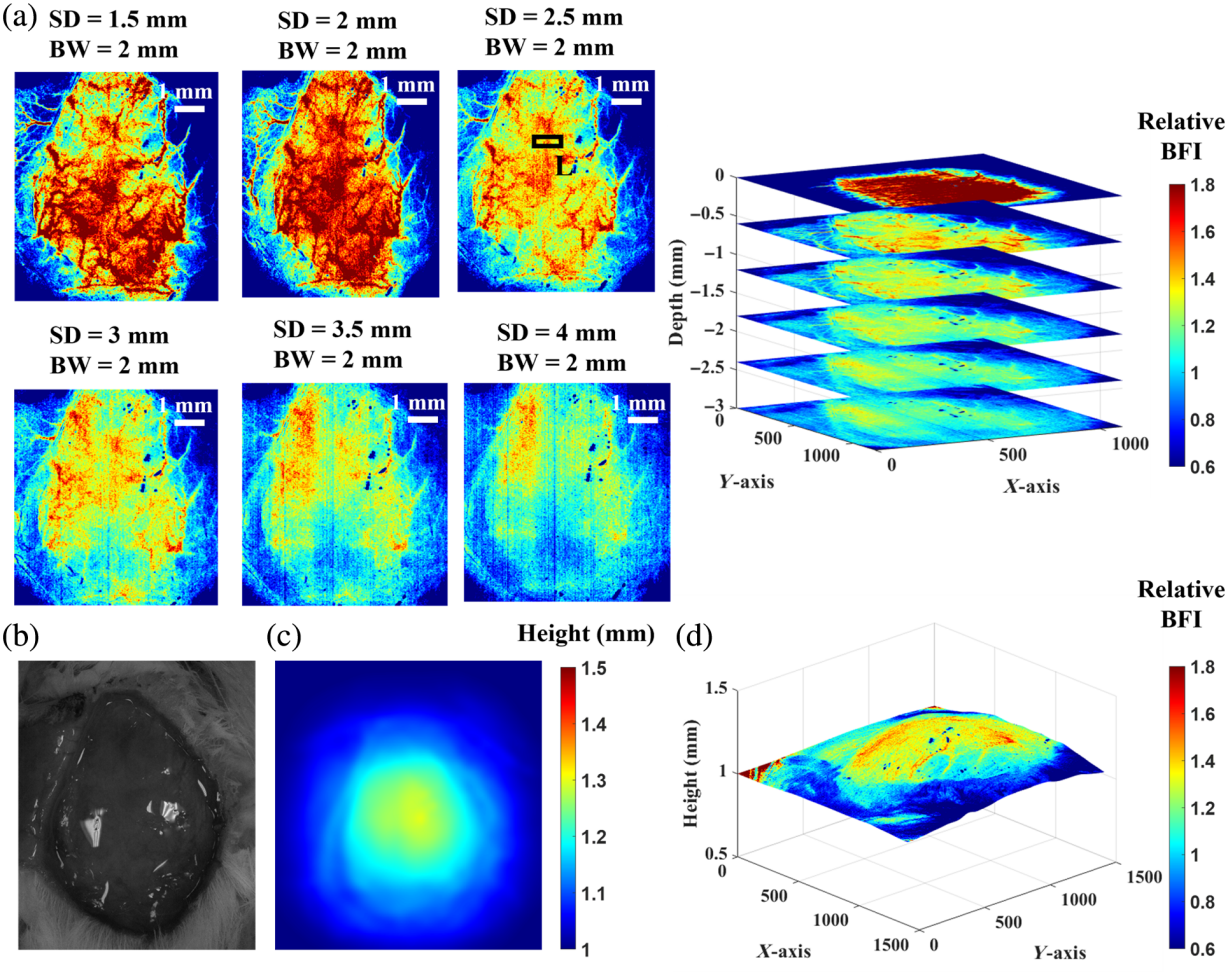
Depth-sensitive imaging of BFI maps from the rat head at baseline. (a) A series of 2D BFI maps reconstructed using SDs ranging from 1.5 to 4 mm with a BW of 2 mm. Spatial resolution was quantified by selecting the ROI on the 2D flow map (SD = 2.5 mm and BW = 2 mm) over a large bundle of superior cerebral veins (L). (b) Photograph of the rat skull. (c) Corresponding height map of the skull. (d) An integrated 2D BFI map overlaid on the 3D head surface geometry.

Figure 8(a) shows depth-sensitive BFI maps of a rat (Table 2) during the baseline measurement. Given that skull thicknesses of adult rats are ∼1  mm,^40^ a series of 2D BFI maps were reconstructed using SDs ranging from 1.5 to 4 mm and a BW of 2 mm. Figures 8(b) and 8(c) show a photograph and corresponding height map of the rat head, respectively. Figure 8(d) presents the integrated BFI map overlaid on the 3D head surface geometry.

Based on the results in Figs. 4 and 8, an SD of 2.5 mm and a BW of 2 mm were selected to capture cortical BFI at depths of 1.5 to 2 mm in the rat. Spatial resolution was then quantified by selecting an ROI over a large vein (L) in Fig. 8(a), with estimated values of ∼300  μm, calculated from pixel counts within the ROI and scaled to the corresponding head dimensions.

**Fig. 9:**
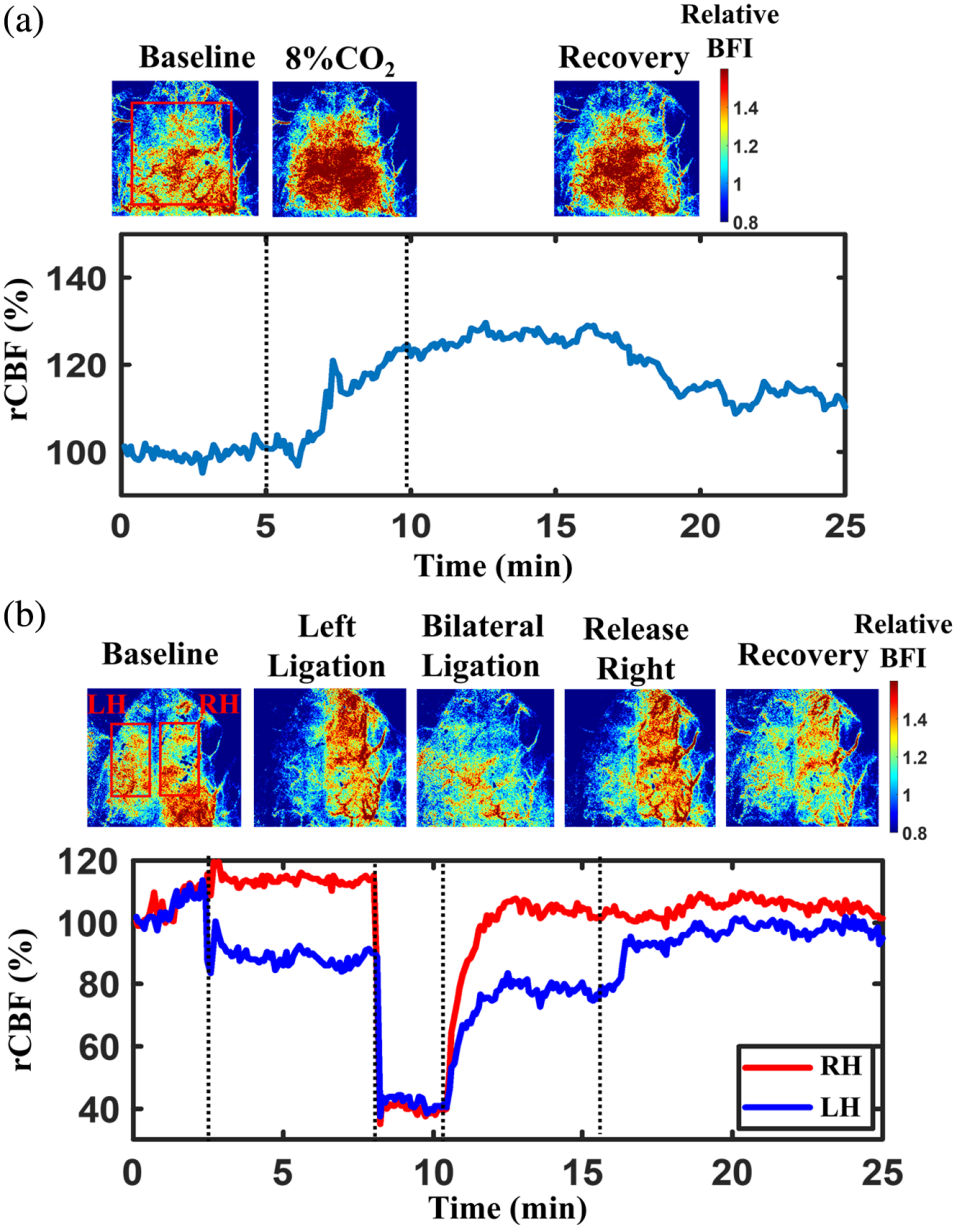
Continuous monitoring of rCBF variations during 8% ${\mathrm{CO}}_{2}$ inhalation and transient carotid artery ligations was performed in a rat with the scalp retracted. (a) BFI maps and corresponding time-course rCBF changes before, during, and after 8% ${\mathrm{CO}}_{2}$ inhalation. Dashed lines indicate the separation of experimental phases, including 5 min of baseline, 5 min of ${\mathrm{CO}}_{2}$ inhalation, and 15 min of recovery. (b) BFI maps and time-course rCBF changes before, during, and after sequential carotid artery ligations. Dashed lines delineate 3 min of baseline, 5 min of left carotid artery ligation, 2 min of bilateral ligation, 5 min following release of the right carotid artery, and 10 min of recovery after release of the left carotid artery. The temporal resolution was ∼2.5  s.

Figure 9(a) shows the time-course changes in rCBF measured before, during, and after ${\mathrm{CO}}_{2}$ inhalation (8% ${\mathrm{CO}}_{2}/92\%$ $O_{2}$). Given the global effect of ${\mathrm{CO}}_{2}$ on cerebral vasculature, time-course rCBF values were calculated by normalizing the mean BFI value within the ROI encompassing the entire brain (indicated by the red rectangular box) to its baseline value preceding ${\mathrm{CO}}_{2}$ exposure.

During ${\mathrm{CO}}_{2}$ inhalation, rCBF increased to 119.7%, reflecting enhanced cerebral perfusion due to hypercapnia-induced vasodilation. In the recovery phase, rCBF gradually returned toward baseline but did not fully normalize (rCBF = 108.3%) within 15 min, consistent with the expected delayed hemodynamic recovery following ${\mathrm{CO}}_{2}$ exposure.

Figure 9(b) shows the time-course rCBF values before, during, and after transient global ischemia, induced through unilateral and bilateral CCA ligations. Two ROIs were selected on the LH and RH, respectively, to assess hemispheric differences in BFI changes over time. rCBF values over time were calculated by normalizing mean BFI values in two hemispheres (LH and RH) at each time point to their respective baseline values prior to CCA ligations.

Notable interhemispheric differences in rCBF were observed across different experimental phases, including left CCA ligation (rCBF in LH = 84.2% and rCBF in RH = 111.3%), bilateral CCA ligation (rCBF in LH = 43.5% and rCBF in RH = 42.8%), release of the right CCA ligation (rCBF in LH = 79.4% and rCBF in RH = 83.4%), and the recovery phase (rCBF in LH = 95.7% and rCBF in RH = 102.3%). These results meet pathophysiological expectations and are consistent with previously reported results in rats using our larger scDCT system.^22^

## 4 Discussion and Conclusion

We present an affordable, user-friendly mini-scDCT for depth-sensitive CBF imaging in small rodents, adapted from a large, costly scDCT prototype originally designed for both small animals and human subjects. The mini-scDCT replaces bulky components with compact, low-cost alternatives while maintaining imaging performance. Mounted on a standard stereotaxic frame, it offers portability and ease of use. Temporal resolution was enhanced through hardware synchronization and software optimization of control through LabVIEW. Compared with the original scDCT, the mini-scDCT achieved a fourfold cost reduction, a fivefold reduction in footprint, and eightfold temporal resolution per source.

Mini-scDCT performance was evaluated using head-simulating phantoms and rodent models under diverse hemodynamic and pathophysiological conditions. In phantom tests, reconstructed 2D flow map quality at varying SDs and BWs was quantified using PSNR. Very short SDs caused detector saturation and narrow BWs left regions unsampled, whereas large SDs produced weak signals and wide BWs reduced depth sensitivity and spatial resolution. These factors all lowered PSNR, highlighting the need to jointly optimize SDs and BWs to improve imaging quality across depths.

Phantom experiments provided practical guidance for optimizing mini-scDCT configurations (SDs and BWs) to achieve high-quality imaging in mice and rats with known cranial geometries. In animal studies, skull thicknesses were considered in selecting the SD and BW to capture brain-specific signals underneath the skull. With the optimized SD and BW, the resulting cortical spatial resolutions were ∼40  μm in mice and ∼300  μm in rats, respectively.

The mini-scDCT’s capability for longitudinal monitoring of global and regional rCBF was evaluated in nine mice divided into stroke and sham groups. In sham mice, rCBF remained stable on day 1 with slight elevations on day 2, likely from postoperative inflammation. In stroke mice, ipsilateral rCBF (LH) dropped by 42.3%±9.1% immediately after induction and remained low (48.2%±9.7% at day 2). Contralateral rCBF (RH) showed a transient increase of 6.8%±15.5%, followed by a decline of 14.7%±12.5% after 10 min, and a further reduction of 31.2%±16.2% by Day 2. These rCBF dynamics (see Fig. 7 and Table S1 in the Supplementary Material) align with physiological expectations: persistently reduced perfusion in the infarcted hemisphere and variable compensatory patterns contralaterally.

Although no identical experimental results are available for direct comparison, our findings are consistent with prior LSCI studies of acute MCAO in stroke mice, which reported CBF reductions of 40% to 85%.^41^^–^^44^ This broad range reflects variations in stroke models, severity, ROI selection, and measurement timing. Our observed reduction (42.3%±9.1%) lies at the lower end, likely for several reasons. First, unlike studies that excluded moderate ischemia using strict thresholds (e.g., >75% CBF reduction^41^^–^^43^ or neurological deficit criteria based on the five-point Longa scale^44^), we included a broader spectrum of responses. Second, although infarct-centered ROIs typically yield larger reductions, we used a standardized rectangular ROI across all animals for consistency (Fig. 6), which may underestimate reductions in smaller infarcts. Finally, technical challenges in MCAO modeling, such as infarct size variability from fixed filament diameters, also contribute to inter-animal variability.^45^

We further assessed the measurement accuracy of the mini-scDCT system in a rat by comparing the results with our previous studies using the original scDCT system under the same experimental protocol.^25^ During 8% ${\mathrm{CO}}_{2}$ inhalation, global rCBF increased to 119.7%, whereas bilateral CCA ligation led to reductions of 43.5% in the LH and 42.8% in the RH (Fig. 9). These findings align with physiological expectations and are highly consistent with previous scDCT studies in rats, which reported a global rCBF increase of 117%±5% during CO_2_ inhalation and regional reductions of 41%±10% in the RH and 37%±9% in the LH during bilateral CCA ligation.^25^

Future improvements may include real-time data analysis, replacement of the costly CrystaLaser with affordable laser diodes, and integration of multiwavelength capability for simultaneous CBF and oxygenation monitoring.^46^ To enable absolute measurements, we plan to adopt recently developed multi-exposure and multidistance approaches for deriving absolute BFI values,^19^^–^^23^ which can then be calibrated against gold-standard modalities such as perfusion MRI.^47^ Geometry correction may also be implemented to further enhance accuracy and functionality.^38^ In addition, point scanning could be replaced with line-shaped scanning to improve temporal resolution.^33^

## Supplementary Material

10.1117/1.JBO.30.10.106007.s01

## Data Availability

All codes and data supporting the findings of this study are available from the corresponding author upon reasonable request.
