# Supplementary material for: Affordable miniaturized speckle contrast diffuse correlation tomography device for depth-sensitive mapping of cerebral blood flow in rodents
Source: J Biomed Opt. 2025 Oct 24;30(10):106007. doi: 10.1117/1.JBO.30.10.106007 (PMC12551968; doi:10.1117/1.JBO.30.10.106007)
Supplement: Supplementary file 1 [file JBO_030_106007_SD001.pdf]

Table S1 Group average rCBF changes (mean ± standard error) from their baseline (100%) in 6 stroke mice and 3 sham mice

Mice	Brain Region	10 minutes	20 minutes	40 minutes	60 minutes	24 hours
**Stroke (n = 6)**	**LH**	57.7% ± 9.1%	56.3% ± 8.1%	52.8% ± 8.0%	56.3% ± 8.2%	52.8% ± 9.7%
	**RH**	106.2% ± 15.5%	92.3% ± 12.5%	89.0% ± 12.2%	91.3% ± 13.8%	69.8% ± 16.15%
**Sham (n = 3)**	**LH**	90.2% ± 1.5%	87.8%f ± 1.9%	89.3% ± 2.5%	94.2% ± 2.4%	103.3% ± 6.0%
	**RH**	95.6% ± 1.5%	98.2% ± 3.1%	98.6% ± 3.9%	97.08% ± 4.3%	101.13% ± 8.5%
